# Transforming glaucoma diagnosis: transformers at the forefront

**DOI:** 10.3389/frai.2024.1324109

**Published:** 2024-01-15

**Authors:** Farheen Chincholi, Harald Koestler

**Affiliations:** Department of Computer Science, Chair of Computer Science 10 (System Simulation), Friedrich-Alexander-Universität Erlangen-Nürnberg (FAU), Erlangen, Germany

**Keywords:** ViT, DETR, object detection, medical imaging, glaucoma

## Abstract

Although the Vision Transformer architecture has become widely accepted as the standard for image classification tasks, using it for object detection in computer vision poses significant challenges. This research aims to explore the potential of extending the Vision Transformer for object detection in medical imaging, specifically for glaucoma detection, and also includes an examination of the Detection Transformer for comparative analysis. The analysis involves assessing the cup-to-disc ratio and identifying signs of vertical thinning of the neuroretinal rim. A diagnostic threshold is proposed, flagging a cup-to-disc ratio exceeding 0.6 as a potential indicator of glaucoma. The experimental results demonstrate a remarkable 90.48% accuracy achieved by the pre-trained Detection Transformer, while the Vision Transformer exhibits competitive accuracy at 87.87%. Comparative evaluations leverage a previously untapped dataset from the Standardized Fundus Glaucoma Dataset available on Kaggle, providing valuable insights into automated glaucoma detection. The evaluation criteria and results are comprehensively validated by medical experts specializing in the field of glaucoma.

## 1 Introduction

Glaucoma, a complex and progressive eye disease, stands as a major public health concern worldwide. It is characterized by the gradual and irreversible deterioration of the optic nerve, typically associated with elevated intraocular pressure (IOP), although glaucoma can also develop with normal IOP. This condition eventually leads to a gradual loss of vision and, if left untreated, can ultimately result in blindness. The impairment typically begins with peripheral vision loss and, if undetected and untreated, can advance to affect central vision as well (Wagner et al., 2022).

Glaucoma is often referred to as the “silent thief of sight” because its early stages usually manifest without noticeable symptoms or pain (National Eye Institute, 2023). Individuals might remain unaware of the disease until significant vision loss has occurred. Given its potential for severe vision impairment and the lack of a cure, glaucoma management focuses on early detection, continuous monitoring, and appropriate treatment to slow down or halt disease progression.

**Optic nerve cupping**

**Figure 1:**
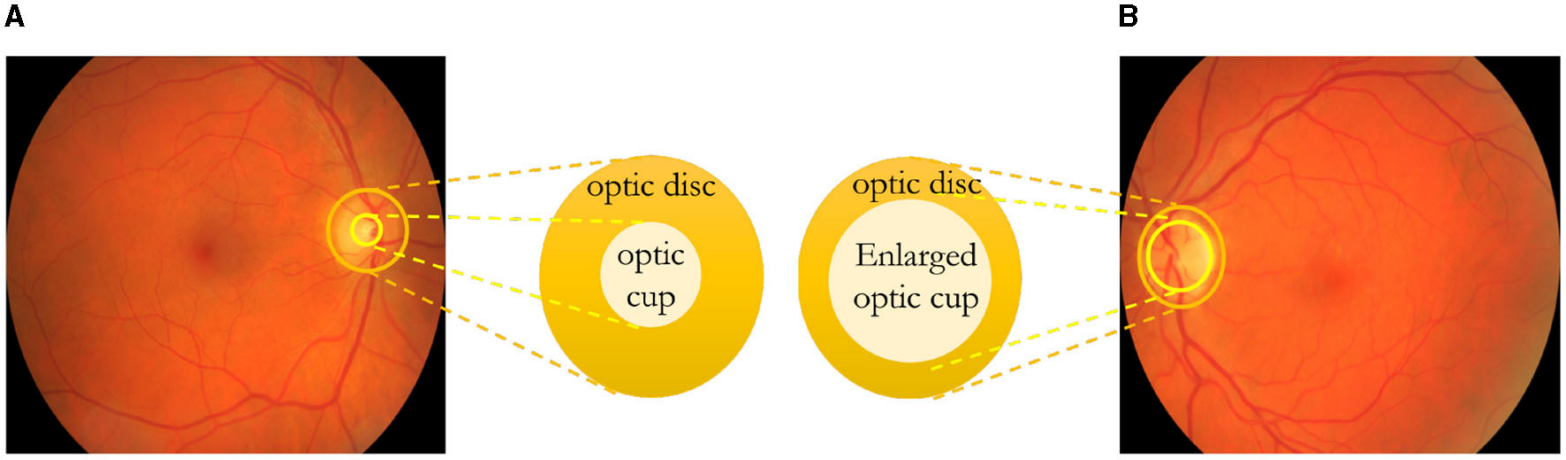
In **(A)**, a healthy eye is depicted, while in **(B)**, optic nerve cupping is illustrated, clearly demonstrating an increase in the cup-to-disc ratio. **(A)** Healthy eye. **(B)** Eye with glaucoma.

The optic nerve transmits visual signals from the eye's retina to the brain (Glaucoma Research Foundation, 2023). It comprises numerous retinal nerve fibers that converge and exit through the optic disc situated at the eye's posterior. The optic disc has a central section known as the “cup,” typically smaller than the entire optic disc depicted in Figure 1A. In individuals with glaucoma, Figure 1B shows that increased eye pressure and/or reduced blood flow to the optic nerve cause the degeneration of these nerve fibers. Consequently, the cup enlarges in relation to the optic disc due to a lack of support. Optic nerve cupping worsens as the cup-to-disc ratio increases. Both individuals with and without optic nerve damage exhibit optic nerve cupping, although those with glaucoma often have a higher cup-to-disc ratio. A cup-to-disc ratio exceeding six-tenths is generally considered suspicious for glaucoma. Regular optic nerve photographs enable monitoring of the cup-to-disc ratio. This assists the doctor in assessing whether nerve fiber damage is ongoing despite current treatment and whether treatment adjustments are necessary.

This paper develops deep learning-based screening software for the detection and location of glaucoma from digital fundus images. In recent advancements, Vision Transformer (ViT) (Dosovitskiy et al., 2020) and the Detection Transformer (DETR) (Carion et al., 2020) models have gained significant traction in the domain of computer vision. ViT, especially, has demonstrated outstanding performance in image classification. This study takes a significant stride by extending the application of ViT to object detection tasks, particularly focusing on its integration into medical imaging for the purpose of glaucoma detection. The research zeroes in on identifying the cup-to-disc (OC to OD) ratio and detecting signs of vertical thinning in the neuroretinal rim (tOD) as key indicators. If the cup-to-disc ratio exceeds six-tenths, the condition is flagged as glaucoma. This approach holds promise for an automated, precise, and efficient process in glaucoma detection. The prior research in this field, encompassing classical machine learning and deep learning, is critically reviewed and discussed in Section 2.

## 2 Related work

### 2.1 Classical machine learning methods

In the study on glaucoma diagnosis (An et al., 2019), the authors developed a machine learning algorithm utilizing optical coherence tomography (OCT) data and color fundus images. Convolutional neural networks (CNNs) with various input types, including fundus images, retinal nerve fiber layer thickness maps, and ganglion cell complex thickness maps, were employed. Through data augmentation and dropout, the CNNs achieved strong performance. To combine CNN model results, a random forest (RF) was trained to classify disc fundus images of healthy and glaucomatous eyes. This classification was based on feature vector representations of each input image, obtained by removing the second fully connected layer. The combined approach demonstrated high accuracy, surpassing individual input methods, with a 10-fold cross-validation area under the receiver operating characteristic curve (AUC) reaching 0.963.

Praveena and GaneshBabu proposed a K-means clustering-based approach to automatically extract the optic disc (Praveena and Ganeshbabu, 2021). The optimal K value in K-means clustering was determined using hill climbing. The optic cup's segmented contour was refined through elliptical and morphological fitting methods. The cup-to-disc ratio was calculated and compared with ophthalmologist-provided values for 50 normal and 50 glaucoma patient fundus images. The mean errors for elliptical and morphological fitting with K-means clustering were 4.5% and 4.1%, respectively. Adopting fuzzy C-means clustering reduced the mean errors to 3.83% and 3.52%. Clustering and segmentation using SWFCM achieved mean error rates of 3.06% and 1.67%. The fundus images were sourced from Aravind Eye Hospital, Pondicherry.

Civit-Masot et al. (2020) have developed a diagnostic aid tool aimed at detecting glaucoma using eye fundus images. The tool is meticulously trained and tested, consisting of two subsystems operating independently and integrating their results to enhance glaucoma detection. In the first subsystem, a blend of machine learning and segmentation techniques is employed to autonomously detect the optic disc and cup. These detections are then combined, and their physical and positional features are extracted. On the other hand, the second subsystem employs transfer learning techniques on a pre-trained convolutional neural network (CNN) to detect glaucoma by analyzing the complete eye fundus images. The outcomes from both subsystems are amalgamated to differentiate positive cases of glaucoma and enhance the final detection results. The research demonstrates that this integrated system attains a superior classification rate compared to previous works, achieving an impressive area under the curve (AUC) of 0.91.

### 2.2 Deep learning methods

Chen et al. (2015) presented early work utilizing deep convolutional neural networks for glaucoma detection. This study introduced a deep learning architecture focusing on automated glaucoma diagnosis using CNNs. The proposed architecture comprised four convolutional layers and two fully-connected layers, demonstrating promising results in discriminating between glaucomatous and non-glaucomatous patterns. The authors utilized techniques like dropout and data augmentation to enhance diagnostic performance. Their method was evaluated on the ORIGA and SCES datasets, achieving significant improvements with an area under the curve (AUC) of the receiver operating characteristic (ROC) curve at 0.831 and 0.887 for glaucoma detection in the respective databases (Chen et al., 2015).

Yu et al. (2019) developed an advanced segmentation method for optic disc and cup using a modified U-Net architecture. This approach leverages a combination of encoding layers from the widely adopted pre-trained ResNet-34 model and classical U-Net decoding layers. The model was meticulously trained on the recently available RIGA dataset, achieving an impressive average dice value of 97.31% for disc segmentation and 87.61% for cup segmentation. These results are comparable to the performance of experts in optic disc/cup segmentation and Cup-Disc-Ratio (CDR) calculation on a reserved RIGA dataset. When evaluated on the DRISHTI-GS and RIM-ONE datasets without re-training or fine-tuning, the model demonstrated performance on par with state-of-the-art methodologies reported in the literature. Furthermore, the authors fine-tuned the model on two databases, achieving outstanding results. For the DRISHTI-GS test set, the average disc dice value was 97.38%, and the cup dice value was 88.77%. In the case of the RIM-ONE database, the model achieved a disc dice of 96.10% and a cup dice of 84.45%. These results represent the state-of-the-art performance on both databases concerning cup and disc dice values.

Al-Bander et al. (2017) investigated the potential of employing deep learning to automatically acquire features and identify signs of glaucoma in colored retinal fundus images. They developed a fully automated system that utilized a convolutional neural network (CNN) to differentiate between normal and glaucomatous patterns for diagnostic purposes. This innovative approach automatically extracted features from the raw images using CNN, diverging from traditional methods that relied on manually crafted optic disc features. The extracted features were then fed into a Support Vector Machine (SVM) classifier to categorize the images as normal or abnormal. The achieved results demonstrated an accuracy of 88.2%, a specificity of 90.8%, and a sensitivity of 85%.

These investigations showcase the efficacy of both traditional machine learning and deep learning approaches in addressing glaucoma detection using digital fundus images. Nevertheless, there is a pressing requirement for additional research aimed at enhancing the precision and resilience of these techniques. Developing artificial intelligence for clinical use in glaucoma faces several challenges.

## 3 Method

The model design incorporates the architecture of both the Vision Transformer and Detection Transformer, shaping the framework for glaucoma detection as outlined in the process in Section 3.3.

### 3.1 Vision transformer or ViT

The Vision Transformer (ViT) Dosovitskiy et al. (2020) is a deep learning architecture designed for computer vision tasks, particularly image classification. It's an innovative approach that applies the transformer architecture, originally developed for natural language processing (NLP), to process and analyze images.

**Figure 2:**
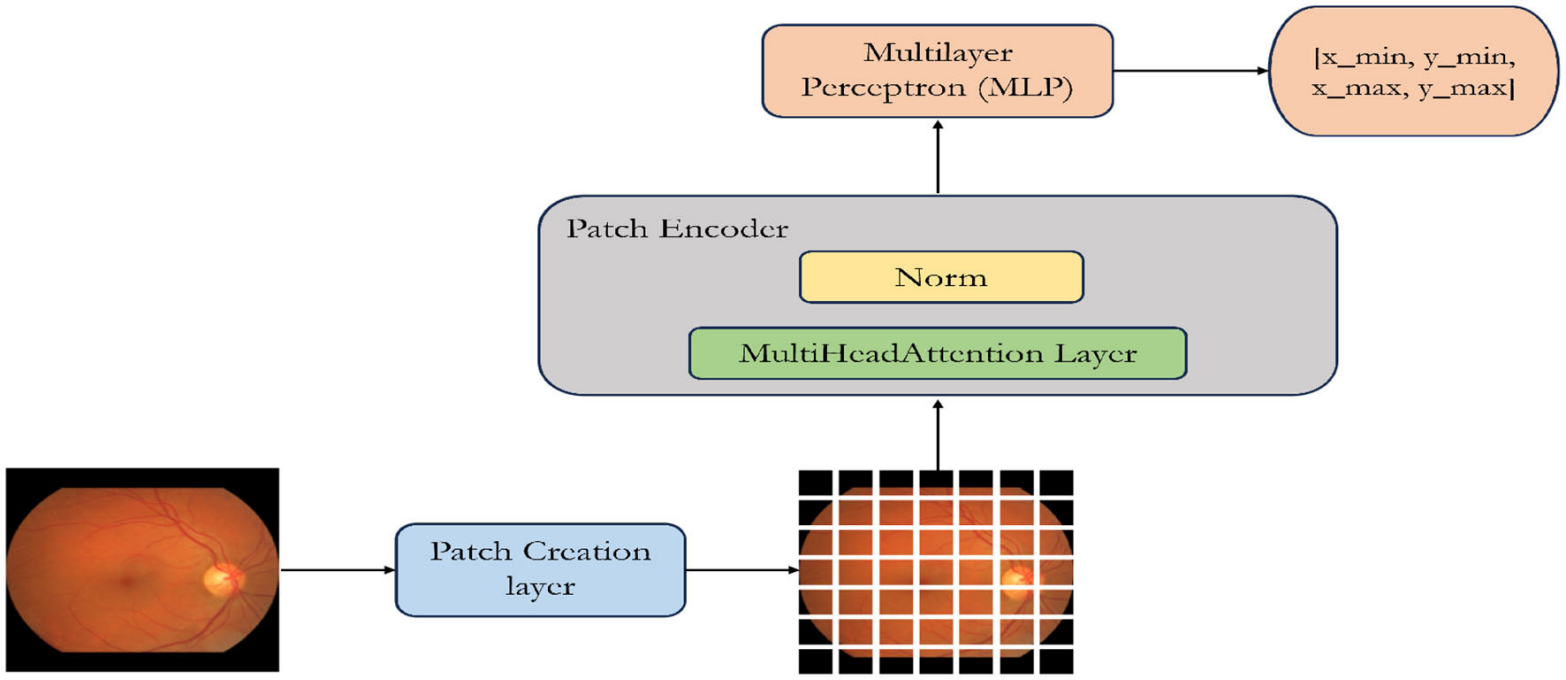
Illustration of a custom-designed Vision Transformer architecture specifically optimized for the detection of optic disc and optic cup.

ViT starts by segmenting the input image into uniform, non-overlapping patches as illustrated in Figure 2. These patches are considered as “tokens” and undergo linear embedding to convert pixel values into higher-dimensional vectors. The patches, now represented as tokens, are essential for capturing image features. To preserve spatial relationships, positional encodings are introduced and added to these token embeddings, indicating their positions in the original image. Next, these token embeddings, along with their positional encodings, are input into the transformer encoder. The transformer comprises multiple layers, each housing two main sub-layers: a multi-head self-attention mechanism and a position-wise fully connected feedforward neural network.

The ViT model is composed of multiple transformer blocks (Vaswani et al., 2017) tailored for object detection. Within each block, the MultiHeadAttention layer facilitates self-attention over the image patch sequence. The “multi-head” concept enables the model to simultaneously learn various attention patterns. The encoded patches (utilizing skip connections) and the outputs from the self-attention layer undergo normalization and are then passed through a multilayer perceptron (MLP) (Taud and Mas, 2018). Following the attention mechanism, position-wise feedforward neural networks process each token independently, integrating insights from the attention mechanism. These operations are performed in each layer of the transformer, enabling the model to comprehend intricate features at different levels of abstraction. The model generates outputs with four dimensions, representing the bounding box coordinates of an object. This design is specifically optimized for object detection tasks, where identifying object positions is a primary objective (Chincholi and Koestler, 2023). The model is trained using labeled data (e.g., image-label pairs) and appropriate loss functions (e.g., cross-entropy loss). The model learns to predict the correct class labels for the input images. ViT offers advantages such as scalability, as it can handle images of varying sizes, and parallelization, which helps in processing patches independently.

### 3.2 DEtection TRansformer or DETR

**Figure 3:**
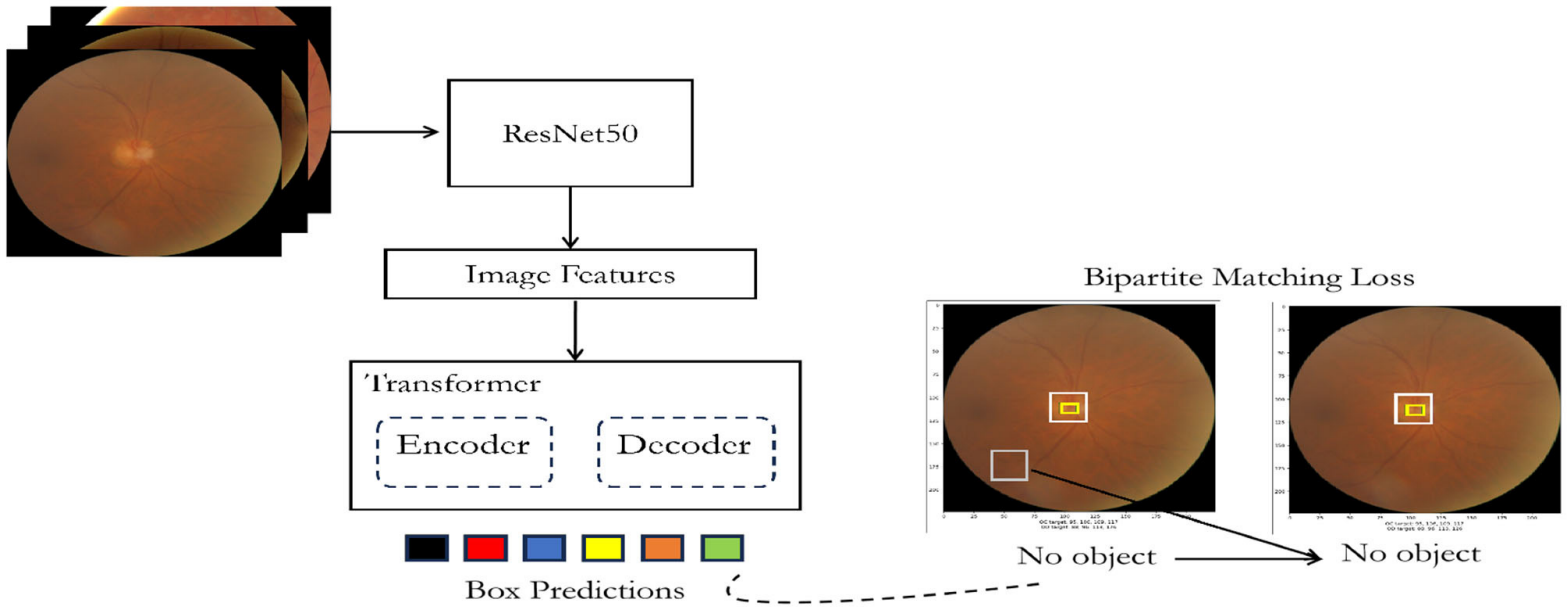
Customized DETR architecture for optic disc and optic cup detection.

The DEtection TRansformer or DETR (Carion et al., 2020) is a transformer-based architecture utilizing an encoder-decoder framework with a convolutional backbone as depicted in Figure 3. The encoder employs a convolutional neural network (CNN) like ResNet (Koonce and Koonce, 2021) to extract feature maps from the input image. Subsequently, the decoder, built on the transformer architecture (Vaswani et al., 2017), processes these feature maps to produce predictions related to objects within the image. To facilitate object detection, two additional heads are integrated into the decoder: a linear layer for handling class labels and a multi-layer perceptron (MLP) for bounding box predictions. The key to object detection lies in utilizing “object queries,” which are responsible for identifying specific objects within an image. In the context of the COCO dataset (Lin et al., 2014), the model employs 100 object queries.

During training, the model leverages a “bipartite matching loss” mechanism. This involves comparing the predicted classes and bounding boxes generated by each of the N (in this case, 100) object queries with the ground truth annotations. The annotations are padded to match the length of N, wherein if an image contains fewer objects (e.g., 4), the remaining annotations (96 in this example) are marked with “no object” for class and “no bounding box.” The Hungarian matching algorithm is employed to establish an optimal one-to-one mapping between the N queries and annotations. Subsequently, standard cross-entropy is utilized for class predictions, and a combined loss comprising L1 and generalized Intersection over Union (IoU) loss is applied for optimizing bounding box predictions.

### 3.3 Process outline

**Figure 4:**
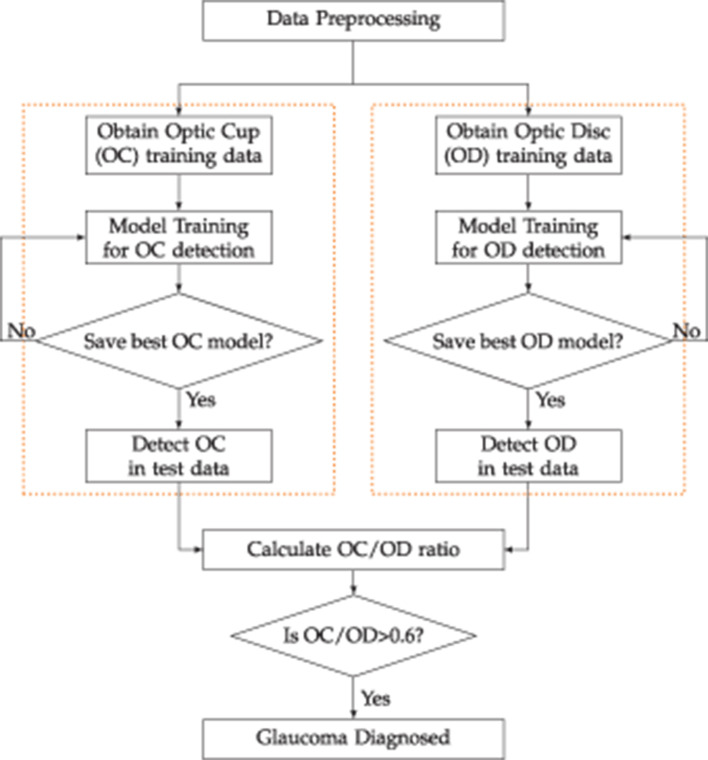
The step-by-step process of detecting optic disc and optic cup using ViT and DETR.

The flowchart in Figure 4 outlines the sequential steps for detecting the Optic Disc (OD) and Optic Cup (OC) using ViT and DETR. It starts with data preprocessing to prepare the input. Following this, the OD data undergoes processing. This involves training the model designed to recognize and pinpoint the optic disc within the given data. The model is instructed and fine-tuned to accurately identify the OD. Subsequently, predictions regarding the location and features of the OD are derived from this trained model.

Simultaneously, the OC data also undergoes a parallel processing trajectory. Similar to the OD data, the OC data is preprocessed and structured for meaningful analysis. A distinct model is then trained to identify the OC accurately. Predictions concerning the OC are generated through this trained model, providing crucial insights into its location and attributes.

The bounding box is defined by its coordinates represented as (*x*_min_, *y*_min_, *x*_max_, *y*_max_), where (*x*_min_, *y*_min_) denotes the top-left corner, and (*x*_max_, *y*_max_) represents the bottom-right corner of the bounding box. To calculate the radius, the distance between the center of the bounding box and one of its corners is computed as follows:


$$\begin{array}{ll} {\text{Center}}_{x} & =\frac{x_{\text{min}}+x_{\text{max}}}{2}\\ {\text{Center}}_{y} & =\frac{y_{\text{min}}+y_{\text{max}}}{2} \end{array}$$

The distance from the center to a corner (for example, the top-left corner) is determined using the Euclidean distance formula:


(1) $$\begin{array}{l} \text{Distance}=\sqrt{{\left({\text{Center}}_{x}-x_{\text{min}}\right)}^{2}+{\left({\text{Center}}_{y}-y_{\text{min}}\right)}^{2}} \end{array}$$

This distance is considered analogous to a radius for a bounding box. An analysis is conducted by assessing the ratio of OC to OD, considering a threshold of 0.6. Depending on this ratio, a diagnosis of glaucoma, is made if the ratio exceeds 0.6. This diagnostic step is critical for identifying and addressing glaucoma in a timely and effective manner.

## 4 Experiments

### 4.1 Datasets

To investigate the scalability of ViT and DETR models, the Standardized Multi-Channel Dataset for Glaucoma (SMDG), a comprehensive open-source resource (Ahalli, 2023; Deathtrooper, 2023) accessible on Kaggle, is utilized. SMDG represents a harmonization effort encompassing 19 public glaucoma datasets, strategically curated to advance AI applications in this domain. This extensive dataset aggregates diverse information, including full-fundus glaucoma images and pertinent image metadata such as optic disc segmentation, optic cup segmentation, blood vessel segmentation, along with any available per-instance textual details such as sex and age.

Remarkably, SMDG-19 stands as the most extensive public repository housing fundus images associated with glaucoma. This research specifically draws on datasets like CRFO-v4, DRISHTI-GS1-TRAIN, DRISHTI-GS1-TEST, G1020, ORIGA-light, PAPILA, and REFUGE1-TRAIN (Retinal Fundus Glaucoma Challenge 1 Train) from SMDG-19 as outlined in Table 1, constituting a crucial aspect of our investigation into ViT and DETR model scalability. Leveraging this diverse and extensive dataset enables a comprehensive exploration of the models' performance and scalability across varied data sources, enriching the findings of our study.

**Table 1:** Overview of utilized datasets sourced from the Standardized Fundus Glaucoma Dataset (SMDG-19) accessible on Kaggle (Ahalli, 2023).

**Dataset**	**CRFO-v4**	**G1020**	**DRISHTI GS1-TRAIN**	**DRISHTI GS1-TEST**	**ORIGA-light**	**PAPILA**	**REFUGE1 -TRAIN**
0 (Non-Glaucoma)	31	724	18	13	482	333	360
1 (Glaucoma)	48	296	32	38	168	87	40

### 4.2 Training and fine-tuning

#### 4.2.1 Preprocessing

For preprocessing during training and validation, images are resized or rescaled so that the shorter side measures at least 224 pixels while the longer side does not exceed 224 pixels. Additionally, they are normalized across the RGB channels.

#### 4.2.2 Computing environment

In the training process of both models, the computing environment employed was the JupyterHub environment running on the Alex cluster.^1^ The cluster consists of a total of 352 Nvidia A40 GPUs, supplemented by 160 Nvidia A100 GPUs with 40GB memory, and an additional 144 Nvidia A100 GPUs with 80GB memory.

#### 4.2.3 Training

DETR was trained using the AdamW optimizer, an extension of the Adam optimizer that incorporates weight decay to prevent overfitting. The training process was facilitated by PyTorch Lightning and the Hugging Face Transformers library.^2^ PyTorch Lightning simplifies the training loop, providing a more modular and readable structure, while the Hugging Face Transformers library offers pre-trained transformer models and tools for various natural language processing tasks. The forward method of the Lightning module was implemented to take pixel values and masks as inputs, generating model predictions—a crucial step in the training process. The initial learning rate for the transformer was set to 1 × 10^−4^, and for the backbone, it was 1 × 10^−5^. Additionally, a weight decay of 1 × 10^−4^ was applied. Transformer weights were initialized using Xavier initialization, a technique that contributes to stable training by appropriately scaling weights based on the number of input and output units in a layer. A ResNet50 backbone, pre-trained on ImageNet—a large-scale dataset of images—was utilized. The training schedule spanned 300 epochs, with each epoch involving a pass over all training images. Hyperparameterization techniques were employed for fine-tuning to optimize the training process.

The ViT model underwent training for 1,000 epochs, utilizing the Adam optimizer with a batch size of 16 and a weight decay of 0.0001, as per Zhang (2018). This weight decay value, contrary to common preference for Stochastic Gradient Descent (SGD), has demonstrated efficacy in model transfer. The training protocol incorporated a linear learning rate set at 0.001, along with a decay strategy. For fine-tuning, SGD with momentum was employed, accompanied by a reduced batch size of 8. The training procedure involved extracting and reshaping patches from input images using a custom Patches Layer, with a patch size of 32 × 32. Each image was divided into 49 patches, resulting in 3,072 elements per patch. The patches were then encoded using learned projections and positional embeddings through the PatchEncoder Layer. Transformer Blocks, consisting of layer normalization, multi-head attention, skip connections, and an MLP with head units configured as [2, 048, 1, 024, 512, 64, 32], facilitated robust feature extraction. The transformer units were characterized by dimensions of projection_dim * 2 and projection_dim, with 4 transformer layers. The Representation Layer flattened the output and applied dropout for enhanced feature representation. Finally, the Bounding Box Output layer produced four neurons representing bounding box coordinates. This comprehensive training approach and architecture, incorporating effective optimization strategies, contribute to the model's performance in object detection tasks.

### 4.3 Optic disc and optic cup detection and localization

**Figure 5:**
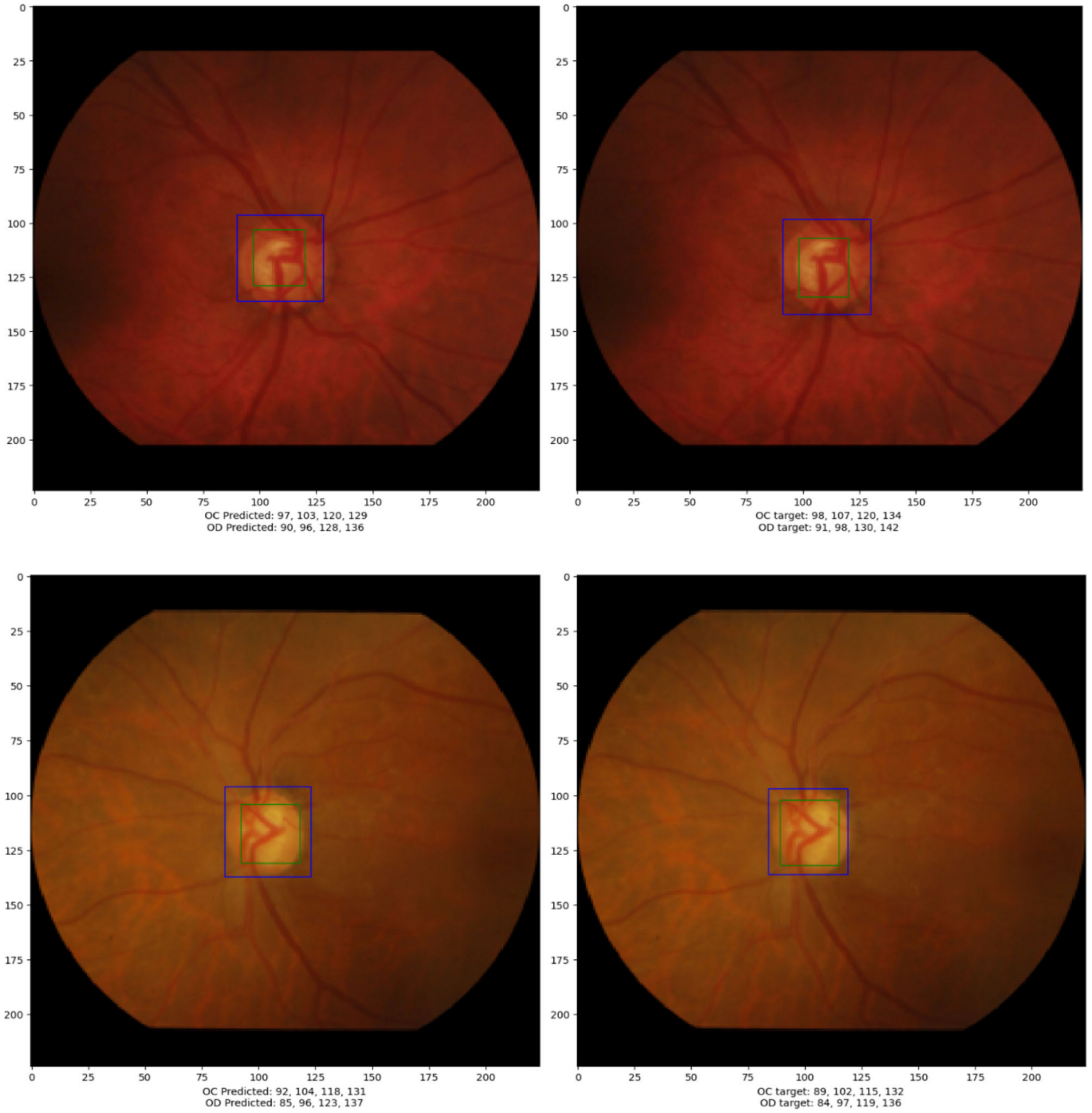
The **left** figure displays the output generated by the models, whereas the **right** figure exhibits the annotated positions of the optic cup (OC) and optic disc (OD).

In this section, a concise evaluation of the detection and localization performance for the optic disc (OD) and optic cup (OC) is provided. Figure 5 visually presents the bounding box coordinates [x_min, y_min, x_max, y_max] representing both the ground truth and predicted objects. These coordinates are obtained by applying the trained model to the test data.

The accompanying table beneath Figure 5 offers an in-depth comparison between the ground truth and predicted bounding box coordinates for both OC and OD for the bottom most image. The table includes calculated radii and the OC/OD ratio. The bounding box coordinates are presented in the format [x_min, y_min, x_max, y_max]. The radii for the optic disc and optic cup are approximate values determined using Equation (1). The OC/OD ratio, calculated as 0.7 in this case, is indicative of a potential diagnosis of glaucoma. This ratio serves as a key metric in evaluating the health of the optic nerve head based on the detected optic disc and optic cup parameters.

Table 2 provides a detailed comparison between ground truth and predicted values for both OD and OC on the test data. Each row corresponds to a specific image (labeled 1 to 9) for both OD and OC. The last section of the table presents calculations, including the OC/OD ratio for ground truth and predictions, along with actual and predicted classifications. This comprehensive evaluation aids in assessing the model's accuracy in predicting OD and OC parameters and is crucial for diagnosing ocular health.

**Table 2:** The table displays both the ground truth and predictions derived from the test data, including the corresponding radius values for each.

	**Image**	**Ground truth**	**Predicted**	**Radius**	
				**Ground truth**	**Predicted**
**OPTIC DISC**	1	[186, 209, 259, 292]	[190, 209, 270, 291]	41.5	41.1
	2	[195, 214, 251, 280]	[201, 217, 258, 280]	33	31.5
	3	[254, 239, 286, 271]	[245, 237, 285, 281]	16	21.9
	4	[231, 236, 275, 277]	[231, 234, 276, 282]	22	24.4
	5	[253, 237, 300, 281]	[250, 240, 291, 282]	23.5	20.6
	6	[227, 239, 267, 276]	[222, 238, 263, 279]	20	20.7
	7	[228, 239, 275, 291]	[223, 242, 277, 295]	26	26.9
	8	[223, 251, 269, 297]	[223, 252, 266, 295]	23	21.6
	9	[200, 228, 285, 315]	[205, 234, 278, 314]	43.5	40.1
**OPTIC CUP**	1	[195. 214. 251. 280.]	[201, 217, 258, 280]	33	31.5
	2	[254. 239. 286. 271.]	[245, 237, 285, 281]	16	21.9
	3	[231. 236. 275. 277.]	[231, 234, 276, 282]	22	24.4
	4	[253. 237. 300. 281.]	[250, 240, 291, 282]	23.5	20.6
	5	[227. 239. 267. 276.]	[222, 238, 263, 279]	20	20.7
	6	[228. 239. 275. 291.]	[223, 242, 277, 295]	26	26.9
	7	[223. 251. 269. 297.]	[223, 252, 266, 295]	23	21.6
	8	[200. 228. 285. 315.]	[205, 234, 278, 314]	43.5	40.1
	9	[200. 216. 285. 304.]	[203, 219, 280, 301]	44	41.1
	**Image**	**Radius**		**Classification**	
		**Ground truth OC/OD**	**Predicted OC/OD**	**Actual**	**Prediction**
**Calculations**	1	0.8	0.8	1	1
	2	0.5	0.6	0	1
	3	0.6	0.6	1	1
	4	0.7	0.5	1	0
	5	0.6	0.6	1	1
	6	0.6	0.6	1	1
	7	0.5	0.5	0	0
	8	0.8	0.8	1	1
	9	0.8	0.7	1	1

Additionally, it provides a thorough calculation of the ratio between the optic cup (OC) and optic disc (OD) (OC/OD).

### 4.4 Comparison of ViT and DETR model results

In this section, a comparison between ViT and DETR will be conducted, focusing on performance evaluation, ROC Curves, and Precision-Recall Curves.

#### 4.4.1 Performance

The Table 3 presents a performance comparison of our object detection models, DETR and ViT, with the state-of-the-art methods discussed in Section 2. For DETR, the model achieved an accuracy of 92.30% on the DRISHTI dataset with IoU of 0.94 and specificity of 0.85. When applied to a broader range of datasets (DRISHTI, CRFO, G1020, ORIGA, PAPILA, REFUGE1), DETR maintained a notable accuracy of 90.48% with IoU of 0.94 and specificity of 0.75.

**Table 3:** Performance comparison of DETR and ViT with state-of-the-art methods. Evaluation metrics include accuracy, sensitivity, specificity, AUC, and ROC.

**Model**	**Datasets**	**Accuracy**	**Sensitivity**	**Specificity**	**AUC**	**ROC**
DETR[ours]	DRISHTI	92.30	0.94	0.85	0.91	0.89
	ALL DATASETS	90.48	0.94	0.75	0.88	0.87
ViT[ours]	DRISHTI	88	1	0.33	0.85	0.80
	ALL DATASETS	87.87	0.92	0.84	0.86	0.82
CNN + RF (An et al., 2019)	OWN DATA	-	-	-	0.96	-
ML+CNN (Civit-Masot et al., 2020)	RIM-ONE V3, DRISHTI	-	-	-	0.91	-
CNN (Chen et al., 2015)	ORIGA and SCES	-	-	-	0.83	0.88
CNN (Al-Bander et al., 2017)	DRISHTI-GS, RIM-ONE, ONHSD	88.20	0.85	0.90	-	-

The results indicate that DETR and ViT demonstrate comparable performance when compared to established methods, highlighting their potential for effective glaucoma detection within the field of medical image analysis.

On the other hand, ViT exhibited an accuracy of 88% with IoU of 1 and specificity of 0.33 on the DRISHTI dataset. When tested on a wider set of datasets (DRISHTI, CRFO, G1020, ORIGA, PAPILA, REFUGE1), ViT showcased an accuracy of 87.87% with IoU of 0.92 and specificity of 0.84. Overall, the results highlight DETR's slightly higher accuracy and specificity compared to ViT across the evaluated datasets. The results suggest that DETR and ViT exhibit competitive performance in comparison to existing methods, showcasing their potential in glaucoma detection in the domain of medical image analysis.

#### 4.4.2 ROC curves

**Figure 6:**
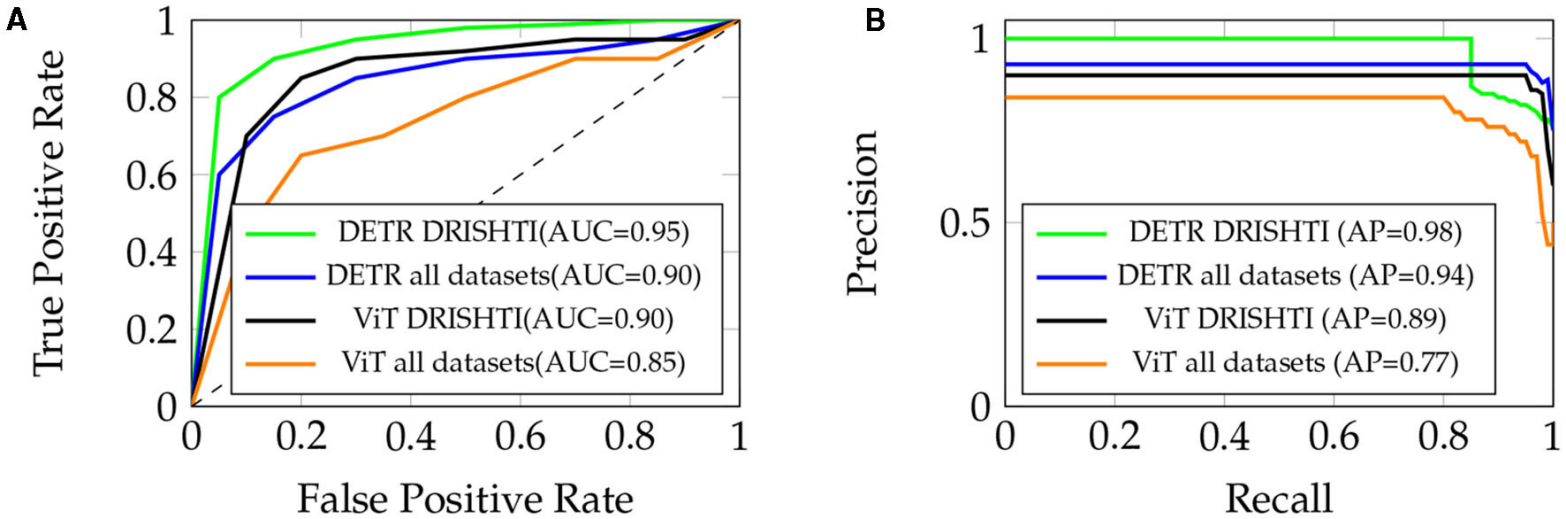
**(A)** ROC Curve for “DETR” and “ViT” models, with AUC of 0.95 and 0.90 respectively (DRISHTI), and 0.90 and 0.95 respectively (all datasets). **(B)** Precision-Recall Curve, with AP of 0.98 (DRISHTI) and 0.94 (all datasets) for “DETR,” and AP of 0.89 (DRISHTI) and 0.77 (all datasets) for “ViT.”

The ROC curve for the “DETR” model highlights its exceptional ability to achieve a high True Positive Rate (sensitivity) even at low False Positive Rates (1-specificity). This is reflected in its impressively high area under the curve (AUC) of about 0.95 for DRISHTI and 0.90 for all datasets, indicating strong discriminative power (refer to Figure 6A). Conversely, the ROC curve for the “ViT” model demonstrates a commendable True Positive Rate at low False Positive Rates, yielding an AUC of approximately 0.90 for DRISHTI and 0.85 for all datasets. Although slightly lower than “DETR,” it still signifies robust model performance.

#### 4.4.3 Precision-recall curves

In the Precision-Recall curve analysis, the “DETR” model showcases excellent precision at relatively high recall values, illustrating its effectiveness in identifying true positive cases while minimizing false positives. In Figure 6B, the area under the curve (AP) for “DETR” is approximately 0.98 for DRISHTI and 0.94 for all datasets, indicating a strong precision-recall trade-off. On the other hand, the “ViT” model displays a reasonable precision-recall trade-off, achieving an AP of approximately 0.89 for DRISHTI and 0.77 for all datasets. This suggests its ability to effectively balance precision and recall.

Both models demonstrate strong discriminative power and a good balance between precision and recall. The ROC and Precision-Recall curves underscore the efficacy of the “DETR” model, particularly in terms of discriminative ability. However, the “ViT” model also performs admirably, striking a commendable balance between precision and recall. The choice of model may depend on specific task requirements, with “DETR” excelling in discriminative power and “ViT” showcasing a favorable precision-recall trade-off.

## 5 Discussion and future work

In conclusion, our investigation into advanced deep learning models, notably the Vision Transformer (ViT) and the Detection Transformer (DETR), for glaucoma detection presents an exciting opportunity to revolutionize this vital medical domain. We have delved into applying Transformers directly to diverse computer vision tasks, including object detection, a pivotal challenge outlined in the original ViT research. By viewing retinal images as sequences of patches and harnessing the power of a Transformer-based architecture, ViT effectively captures intricate patterns and features critical to glaucoma detection, devoid of image-specific biases. This hints at its promising role in automating glaucoma diagnosis. Consequently, ViT either matches or surpasses the state of the art on numerous image classification datasets, all while being relatively cost-effective to pre-train.

On a different note, DETR, tailored for object detection, demonstrates significant potential in precisely localizing specific features within retinal images indicative of glaucoma. This capability enables the accurate detection of affected regions. Its end-to-end detection approach aligns well with the imperative of precisely identifying glaucomatous areas, contributing to heightened diagnostic accuracy. DETR boasts a straightforward implementation and a flexible architecture that can be easily extended to object detection in medical imaging, yielding competitive results.

While these preliminary findings are promising, the development of AI for clinical practice in glaucoma faces significant challenges. Standardizing diagnostic criteria is crucial given the absence of a universally accepted definition for glaucoma. The wide spectrum of the disease and the shortage of glaucoma experts globally contribute to variations in diagnosis and treatment criteria, hindering the development of diagnostic devices. Additionally, collecting detailed data on neurodegenerative and systemic metabolic conditions alongside glaucoma data is crucial for predicting progression. Multifactorial associations between glaucoma and diseases like diabetes and hypertension emphasize the need for AI technology to reveal intricate relationships between systemic conditions and retinal images. To address data shortages and biases, generative AI techniques such as generative adversarial networks (GAN) and emerging diffusion models offer solutions. These models provide data augmentation by generating realistic fundus photographs, overcoming challenges associated with imbalanced medical data. As these generative AI techniques continue to evolve, they hold the promise of synthesizing realistic fundus images based on improved data quality, advancing the field of glaucoma diagnosis and treatment.

Another major hurdle is the ongoing exploration of self-supervised pre-training methods. Our initial experiments have shown improvements from self-supervised pre-training, but a substantial gap persists between self-supervised and large-scale supervised pre-training. Furthermore, scaling up ViT and DETR is likely to lead to enhanced performance. Additionally, a significant challenge lies in further optimizing these models to cater to the unique intricacies of glaucoma detection, such as subtle structural changes in the optic nerve head and retinal nerve fiber layer. Customizing ViT and DETR to extract and interpret features specific to glaucoma pathology is essential for enhancing their effectiveness and reliability in a clinical context.

## Data availability statement

Publicly available datasets were analyzed in this study. This data can be found here: https://www.kaggle.com/datasets/deathtrooper/multichannel-glaucoma-benchmark-dataset.

## Author contributions

FC: Conceptualization, Data curation, Formal analysis, Investigation, Methodology, Resources, Software, Validation, Visualization, Writing – original draft, Writing – review & editing. HK: Project administration, Supervision, Writing – review & editing.

## Footnotes

^1^ https://hpc.fau.de/systems-services/documentation-instructions/clusters/alex-cluster

^2^ https://huggingface.co/facebook/detr-resnet-50
